# Ionothermal Synthesis of Crystalline Nanoporous Silicon and Its Use as Anode Materials in Lithium-Ion Batteries

**DOI:** 10.1186/s11671-019-3024-9

**Published:** 2019-06-06

**Authors:** Fei Wang, Baoxun Zhao, Wenwen Zi, Hongbin Du

**Affiliations:** State Key Laboratory of Coordination Chemistry, School of Chemistry and Chemical Engineering, Nanjing University, Nanjing, 210023 China

**Keywords:** Silicon, Anode material, Nanomaterial, Lithium-ion battery

## Abstract

Silicon has great potential as an anode material for high-performance lithium-ion batteries (LIBs). This work reports a facile, high-yield, and scalable approach to prepare nanoporous silicon, in which commercial magnesium silicide (Mg_2_Si) reacted with the acidic ionic liquid at 100 °C and ambient pressure. The obtained silicon consists of a crystalline, porous structure with a BET surface area of 450 m^2^/g and pore size of 1.27 nm. When coated with the nitrogen-doped carbon layer and applied as LIB anode, the obtained nanoporous silicon-carbon composites exhibit a high initial Coulombic efficiency of 72.9% and possess a specific capacity of 1000 mA h g^−1^ at 1 A g^−1^ after 100 cycles. This preparation method does not involve high temperature and pressure vessels and can be easily applied for mass production of nanoporous silicon materials for lithium-ion battery or for other applications.

**Electronic supplementary material:**

The online version of this article (10.1186/s11671-019-3024-9) contains supplementary material, which is available to authorized users.

## Introduction

The rapidly increasing consumption and high dependence on fossil energy in contemporary society have caused a growing sense of unease about the environment, climate, and energy supply. There is a pressing demand for developing sustainable, portable high-energy and high-power-density energy devices and systems to resolve the temporal energy source and environment mismatch for modern lifestyles [1]. Rechargeable lithium-ion batteries (LIBs) hold remarkable promise for energy storage devices owing to their relatively high energy density and long cycle stability [2, 3]. To meet the increasing requirements of high-performance LIBs, various high-capacity electrode materials are being extensively developed, such as porous amorphous carboneous materials [4, 5], phosphorus-based composites [6, 7], silicon-based composites [8], and transition metal oxides [9, 10]. As a vital component, silicon (Si) is one of the most impressive anodic materials because of its large theoretical capacity (4200 mAh g^−1^), abundant natural sources and relatively safe Li-uptake voltage [11]. Nevertheless, the large-scale practical commercialization of silicon anodic material is plagued by two intricate problems. On the one hand, the enormous volumetric expansion and contraction in the charge and discharge processes lead to the breakdown of the silicon active material, rapid irreversible capacity fading of the battery [12]. On the other hand, the low intrinsic electroconductivity (1.6 × 10^−3^ S/m) of elemental silicon also greatly impedes electron transfer and decreases the rate capability of the electrode.

Recently, considerable efforts have been focused on circumventing the above-mentioned stability issues [13]. A large number of nanostructured silicon materials including nanotubes [14], nanowires/nanorods [15, 16], and nanosheets [17–19] have been engineered to achieve improved structural integrity and cycle performance. Additionally, preparing Si-based porous composites is also considered as an effective method, because appropriate pore spaces in porous silicon composites could act as buffers to mitigate the volume expansion and thereby improve cycling performance in LIBs [20, 21]. For example, Kim et al. fabricated a three-dimensional porous silicon particles by thermal annealing and etching butyl-capped Si gels and SiO_2_ nanoparticles at 900 °C under an Ar atmosphere, which exhibited a stable capacity of over 2800 mA h g^−1^ after 100 cycles at 1 °C [22]. An et al. reported a green, scalable, and controllable pathway to prepare nanoporous silicon (NP-Si) with excellent electrochemical properties from commercial Mg_2_Si alloy via high-temperature vacuum distillation [23]. Though tremendous strides in the consummate electrochemical performance have been demonstrated, most of the preparation methods for these nanoporous structures of Si are generally too complicated to scale up.

Another effective tactic to boost the electrochemical performance of the silicon anode is coating electronically conductive carbon on nanosilicon particles to form silicon-carbon nanocomposites [19, 24], such as yolk-shell [25], watermelon [26], and hollow structures [27]. For instance, Pan et al. designed yolk-shell–structured Si–C nanocomposites with high specific capacity and good cycling stability by a simple and low-cost method based on NaOH etching technology [28]. Chen et al. developed a core-shell–structured Si/B_4_C composite with graphite coating and demonstrated that such composites possessed good long-term cycling stability [29]. Various studies demonstrated that the conductive carbon could not only make up the low electrical conductivity of silicon, but also serve as an elastic intermediary to retard the large volume change and prevent the direct contact between silicon active materials and the electrolyte, leading to enhanced cycling stability [30].

To date, the synthetic routes to silicon nanoparticles (Si NPs) or porous silicon (pSi) usually involve thermal decomposition of silanes [31], chemical etching of Si wafers, and magnesiothermic reduction of SiO_2_ templates [32, 33]. These preparations generally require several steps, high temperature, relatively high-cost templates, etc., which lead to high cost and difficulties to scale up [34]. Recently, the preparation of Si NPs in solution has also been paid much attention [35, 36]. For instance, Kauzlarich et al. reported that SiCl_4_ reacted with NaSi or KSi in organic solvents to obtain silicon nanoparticles [37]. Liang et al. prepared the nest-like silicon nanospheres via a solvothermal reaction, in which NaSi reacted with NH_4_Br in the pyridine and dimethoxyethane mixed solvent in an autoclave at 80 °C for 24 h [38]. The reported solution synthesis generally involved highly active reducing agents such as alkaline metals, LiAlH_4_, and NaSi and often produced low yields or small quantities of Si NPs. In this regard, for mass fabrication of nanosilicon, a low-cost, scalable, and simple approach is still imperative. Herein, we present a convenient, high-yield preparation of porous silicon by oxidation of Mg_2_Si in acidic ionic liquid at 100 °C and ambient pressure. When coated with a nitrogen-doped carbon layer and served as anode of lithium-ion battery, the obtained nanoporous silicon-carbon composites exhibited a high initial Coulombic efficiency (CE) of 72.9% and delivered a specific capacity of 1000 mA h g^−1^ after 100 cycles at 1 A g^−1^.

## Methods

### Materials

1-Butyl-3-methylimidazolium chloride ([Bmim]Cl) was provided by Shanghai Cheng Jie Chemical Co. LTD. Aluminum chloride (AlCl_3_) was purchased from Sinopharm Chemical Reagent Co., Ltd. Magnesium silicide (Mg_2_Si) and commercial silicon powder (1–5 μm) were bought from Alfa Aesar. Battery-grade ethylene carbonate (EC), diethyl carbonate (DEC), fluoroethylene carbonate (FEC), and LiPF_6_ were purchased from Shenzhen Kejingstar Technology Ltd., China. All of the chemicals and reagents were used directly as received.

### Synthesis of Porous Silicon Nanoparticles (pSi)

In a typical procedure, [Bmim]Cl (1.5 g) and AlCl_3_ (4.5 g) with a molar ratio of ~ 1:4 were mixed and loaded in a Schlenk glass tube. Subsequently, 500 mg of magnesium silicide (Mg_2_Si) were added into the glass tube and vigorously agitated at 100 °C for 10 h. The above procedure was conducted in a glovebox filled with Ar. After cooling down, the precipitate was collected and washed with 1 M hydrochloric acid, distilled water, and ethanol. Finally, the product (150 mg, 82% yield) was dried in vacuum for further characterization.

### Synthesis of Nitrogen-Doped Carbon Coated on Porous Silicon Nanoparticles (pSi@NC)

The preparation procedure is referred to the reported literatures [39, 40]. First, 0.1 g of the obtained porous silicon nanoparticles (pSi) were dispersed into 250 mL of deionized water containing sodium dodecylbenzenesulfonate (SDBS; 5 mg) by ultrasonication for 30 min. The mixture was vigorously agitated for 1 h at room temperature. After that, 200 μL of pyrrole monomer, 0.34 g of (NH_4_)_2_S_2_O_8_, and 1.25 mL of 1 M HCl were added into the above solution. After the mixture was stirred in an ice/water bath for 24 h, the formed black powders (denoted as pSi@PPy) were gathered by filtration, washed with deionized water, and dried in vacuum. Finally, the pSi@PPy sample was heated at a ramp rate of 5 °C min^−1^ in a tube furnace to 700 °C for 3 h in a flowing Ar atmosphere to obtain the pSi@NC composite. The carbon content was estimated by thermogravimetric studies.

### Electrochemical Measurements

The electrochemical properties of porous silicon nanoparticles were studied by using a half CR2032 coin cell, in which lithium metal foils served as counter electrodes and reference electrode, the as-prepared pSi@NC as working electrode, polypropylene macroporous films (Celgard 2400) as separators, and 1.0 M LiPF_6_ in 1:1 (v/v) mixture of ethylene carbonate (EC)/diethyl carbonate (DEC) as the electrolyte. The CR2032 cells were assembled in a glovebox with argon atmosphere (oxygen and water contents less than 0.1 ppm). The working anode electrodes were prepared by mixing the obtained pSi@NC composite, super P carbon, and sodium alginate in a weight ratio of 70:20:10 in deionized water to form a homogeneous slurry. Next, the slurry was coated onto Cu foil and dried under vacuum condition at 80 °C for 12 h. The total loading mass of the active materials on the electrode was approximately 0.5 mg cm^−2^. The charge-discharge cycles of the half-cells were performed on a Neware battery tester (Shenzhen, China) at a constant current mode over the range of 0.01–1.5 V. Cyclic voltammetry (CV) of the as-prepared anodes was measured on a CHI650d electrochemical workstation (Shanghai Chenhua Instruments Inc., China), using a three-electrode cell with the voltage sweep rate of 0.2 mV s^−1^ at room temperature. The specific capacity was calculated based on the total mass of the pSi@NC composites.

### Characterization Methods

Power X-ray diffraction (PXRD) measurements were carried out on a Bruker D8 ADVANCE X-ray diffractometer (Cu Kα radiation, 40 kV, 40 mA, *λ* = 1.5418 Å). The morphology and microstructure of the samples were obtained by scanning electron microscopy (Hitachi field-emission scanning electron microscope, S-4800), and the energy-dispersive X-ray spectroscopy was used to analyze the elemental distribution. Transmission electron microscopy (TEM) and high-resolution TEM images were recorded on a JEM-2100 equipment. The porous parameters were determined using a Micromeritics ASAP 2020 analyzer at 77 K after degassing of the sample at 150 °C for 10 h. The specific surface area was calculated using the multiple-point Brunauer−Emmett−Teller (BET) method, and the pore size distribution was analyzed by the density functional theory (DFT) method based on the adsorption data. Raman spectroscopy (LabRAM Aramis, Horiba, equipped with a 633-nm wavelength laser) was used to investigate the structure of nanoporous silicon, which was first calibrated with a Si wafer (520 cm^−1^). PHI 5000 VersaProbe spectrometer was used for X-ray photoelectron spectroscopy (XPS) measurements. Thermogravimetric analysis (TGA) was conducted on a simultaneous STA449F3 (Netzche) thermal analyzer under air atmosphere at 10 °C min^−1^ from 30 to 800 °C in air flowing. Cyclic voltammetry (CV) tests were performed on a CHI650d electrochemical station (Shanghai Chenhua Instruments Inc., China).

## Results and Discussion

**Scheme 1:**
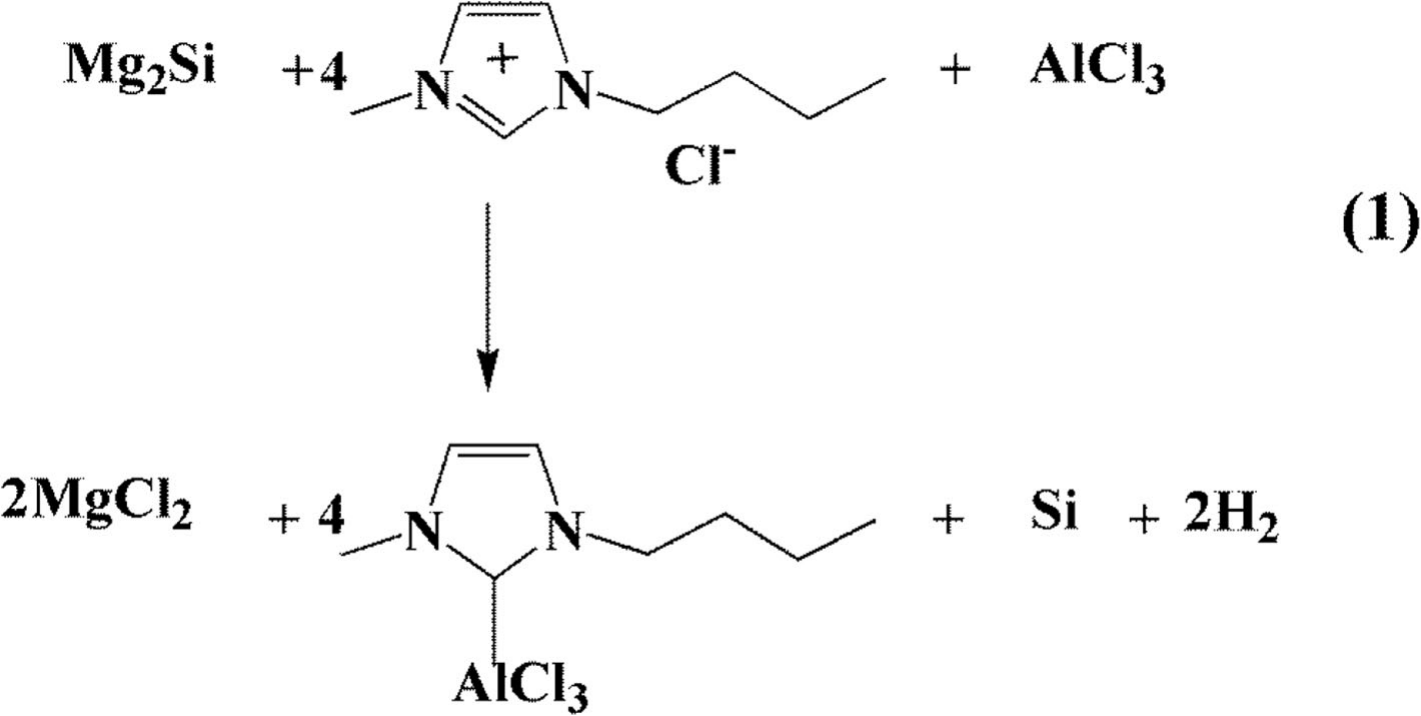
The proposed reaction for preparing pSi

The preparation of porous silicon nanoparticles (pSi) from Mg_2_Si in ionic liquid can be expressed as Reaction 1, as showed in Scheme 1. To understand the reaction process, the pristine products of the proposed Reaction 1 without any washing treatment were directly collected and analyzed by PXRD (Additional file 1: Figure S1). PXRD analysis showed that the pristine product was mainly composed of crystalline Si, by-product inorganic salts MgCl_2_, and reactants Mg_2_Si and AlCl_3_. In the process of preparing porous silicon nanoparticles, 1-butyl-3-methylimidazol chloride and aluminum trichloride at a molar ratio of 1:4 were mixed to ensure that the reaction system is acidic. Then, Mg_2_Si reacted with the acidic system to form the silicon nanoparticles. The yield of porous silicon nanoparticles was over 82% based on the amount of Si atoms in Mg_2_Si. The reaction was carried out in a flask, affording an easily scaling-up, mass production of pSi. The use of ionic liquid [BmimCl]-AlCl_3_ was necessary for the preparation of pSi. Without AlCl_3_, the reaction of Mg_2_Si with [BmimCl] could not take place. Similarly, the Mg_2_Si could not react with AlCl_3_ alone or in other organic solvents such as tetrahydrofuran to produce pSi. We noted that pSi had been previously prepared via thermal decomposition of silanes or silicon halides at high temperature, or their reactions with highly-active reducing agents such as alkaline metals, LiAlH_4_, and NaSi [37, 41]. The use of Mg_2_Si in the preparation of nanostructured silicon by distilling off Mg at high temperature has also been known [23, 42, 43]. However, those reactions often produced low yields or small quantities of pSi. In contrast, the method reported in this work is applicable for mass production of pSi.

**Fig. 1:**
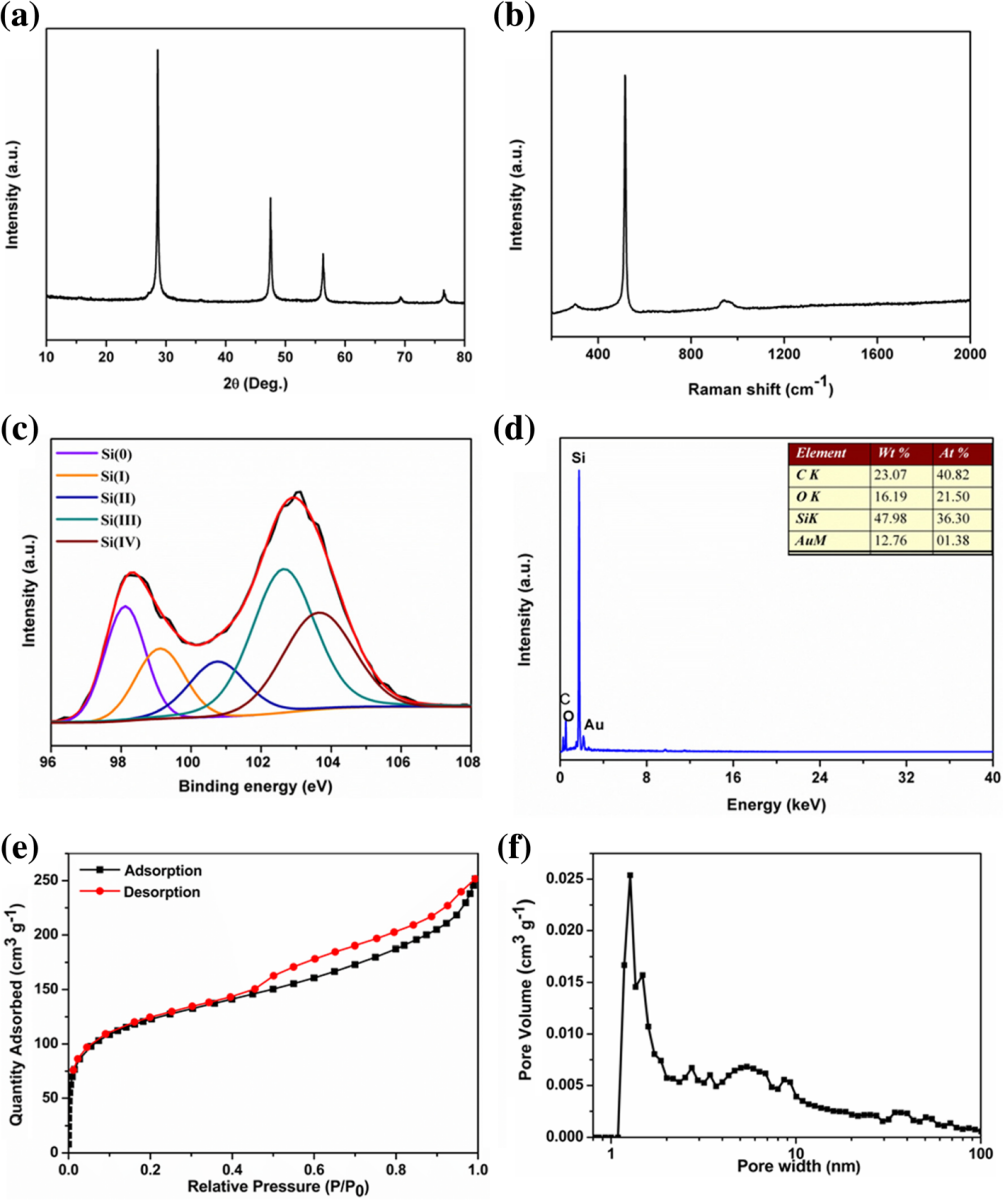
**a** PXRD patterns, **b** Raman spectra, **c** XPS spectrum, **d** EDS spectrum, **e** nitrogen adsorption-desorption curves, and **f** the pore size distribution curve of pSi

The PXRD pattern of the product is shown in Fig. 1a. These five narrow and sharp peaks at 2θ 28.4, 47.3, 56.1, 69.1, and 76.4° are assigned to the (111), (220), (311), (400), and (331) lattice planes of the cubic silicon phase (JCPDS No. 27-1402), which suggests that the obtained silicon is highly crystalline. The average crystallite sizes of the obtained silicon particles were about 40 nm based on Scherrer’s equation. Figure 1b depicts the Raman spectra of the silicon nanoparticles. The typical characteristic peak located at around 518 cm^−1^ corresponds to the Si-Si stretching mode of crystalline Si. The broad band between 900 and 1050 cm^−1^ should be attributed to the second-order spectrum of silicon [44]. And the small peak at ~ 303 cm^−1^ was ascribed to the surface oxide. The specific surface areas and porosity characterization of the obtained samples were elucidated by N_2_ adsorption/desorption isotherms at 77 K. The pSi sample displayed type-IV(a) isothermal sorption curves with a hybrid H2(b)/H3 hysterisis loop, which is characteristic of a porous structure material [45]. It possessed a high Brunauer−Emmett−Teller (BET) surface area of 450 m^2^ g^−1^. The pore size distribution analysis based on DFT method showed that the product consisted of a relatively narrow micropores (1.27 nm) and mesopores (5.4 nm) with broad pore size distribution. The presence of these pores might facilitate Li^+^ ion diffusion.

**Fig. 2:**
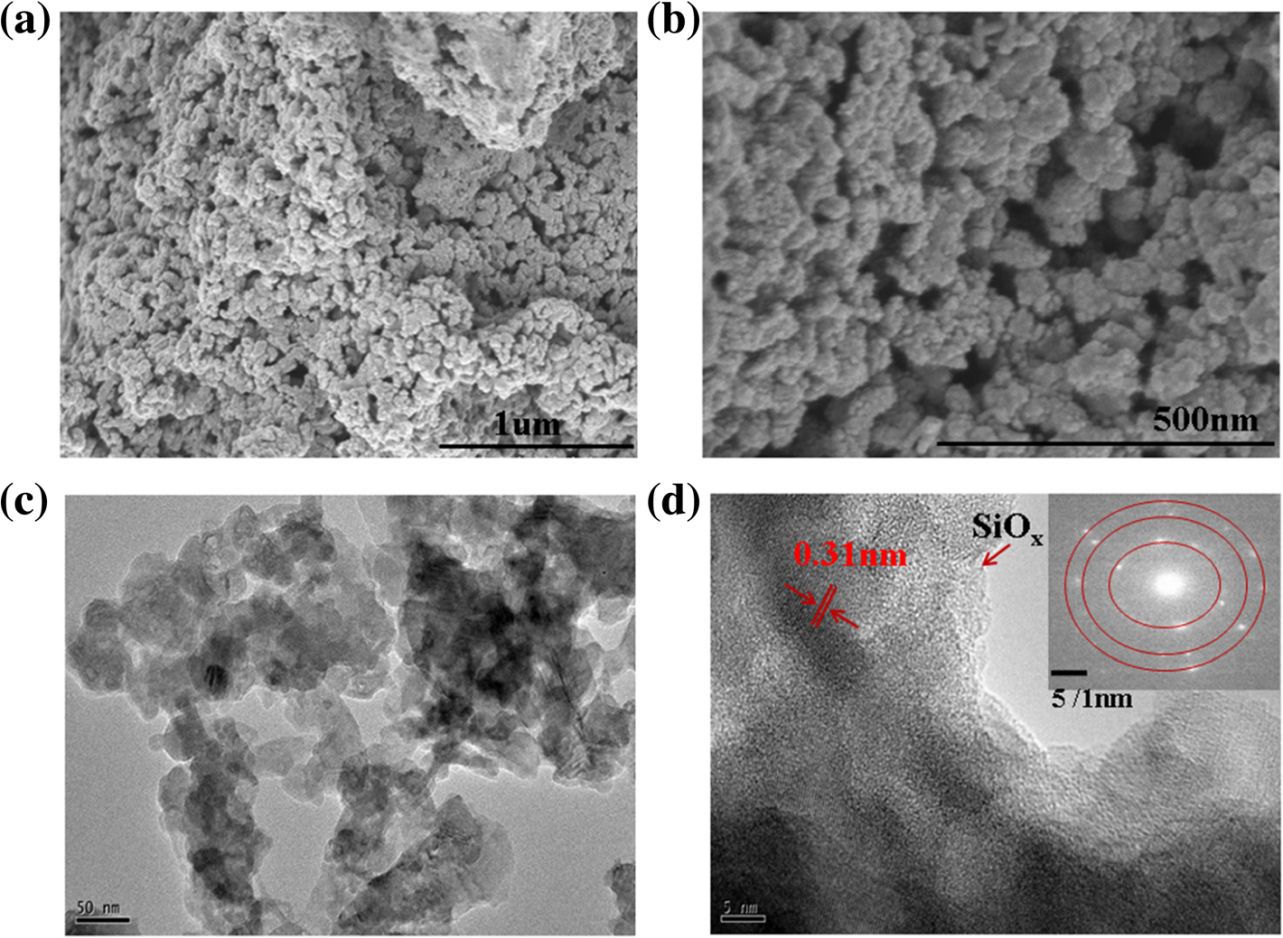
**a**, **b** SEM images and **c**, **d** TEM images of pSi (inset in d shows SAED patterns)

The morphology of the obtained silicon samples was studied by using scanning electron microscopy (SEM) and transmission electron microscopy (TEM). The SEM (Fig. 2a, b, Additional file 1: Figure S2) and TEM (Fig. 2c, d) images show that the overall particle sizes of the obtained nanoporous silicon particles range from several tens to about 100 nm in diameter. The TEM image in Fig. 2c shows that the sample is composed of interconnected silicon particles, resulting in a porous structure. We postulated that the closely arranged Si^4−^ in the micron-sized precursor Mg_2_Si reacted with acidic ionic liquid to form Si surrounded by MgCl_2_ nanoparticles. The latter were washed away by diluted HCl, leaving interconnected pSi with vacancies. The obtained pSi showed a large BET surface area of 450 m^2^ g^−1^ with uniform pore size distribution at 1.27 nm, supporting the above postulation. The HRTEM image of pSi in Fig. 2d reveals that the clear lattice fringe with a typical *d*-spacing of 0.31 nm, attributed to the (111) crystal planes of the cubic Si, is in good agreement with the PXRD results. The interconnected silicon nanoparticles are shown to be covered by a thin oxide layer on the outer surface, owing to the oxidation. The surface composition and valence status of Si nanoparticles were identified by energy-dispersive (EDS) analysis and X-ray photoelectron spectroscopy (XPS). The Si 2p XPS spectrum (Fig. 1c) showed two broad, overlapped peaks at 98.2 eV and 103.0 eV. The two peaks could be divided into five components at 98.11, 99.11, 100.75, 102.64, and 103.64 eV, which were assigned to Si(0), Si(I), Si(II), Si(III), and Si(IV), respectively. The presence of a strong Si(0) peak implies the formation of porous silicon. The stronger Si(III) and Si(IV) peaks suggest the surface of porous silicon was coated by silicon oxide [46]. Consistently, the energy-dispersive (EDS) analysis of pSi showed that the atomic ratio of Si/O on the surface was about 3:2 (Fig. 1d).

**Fig. 3:**
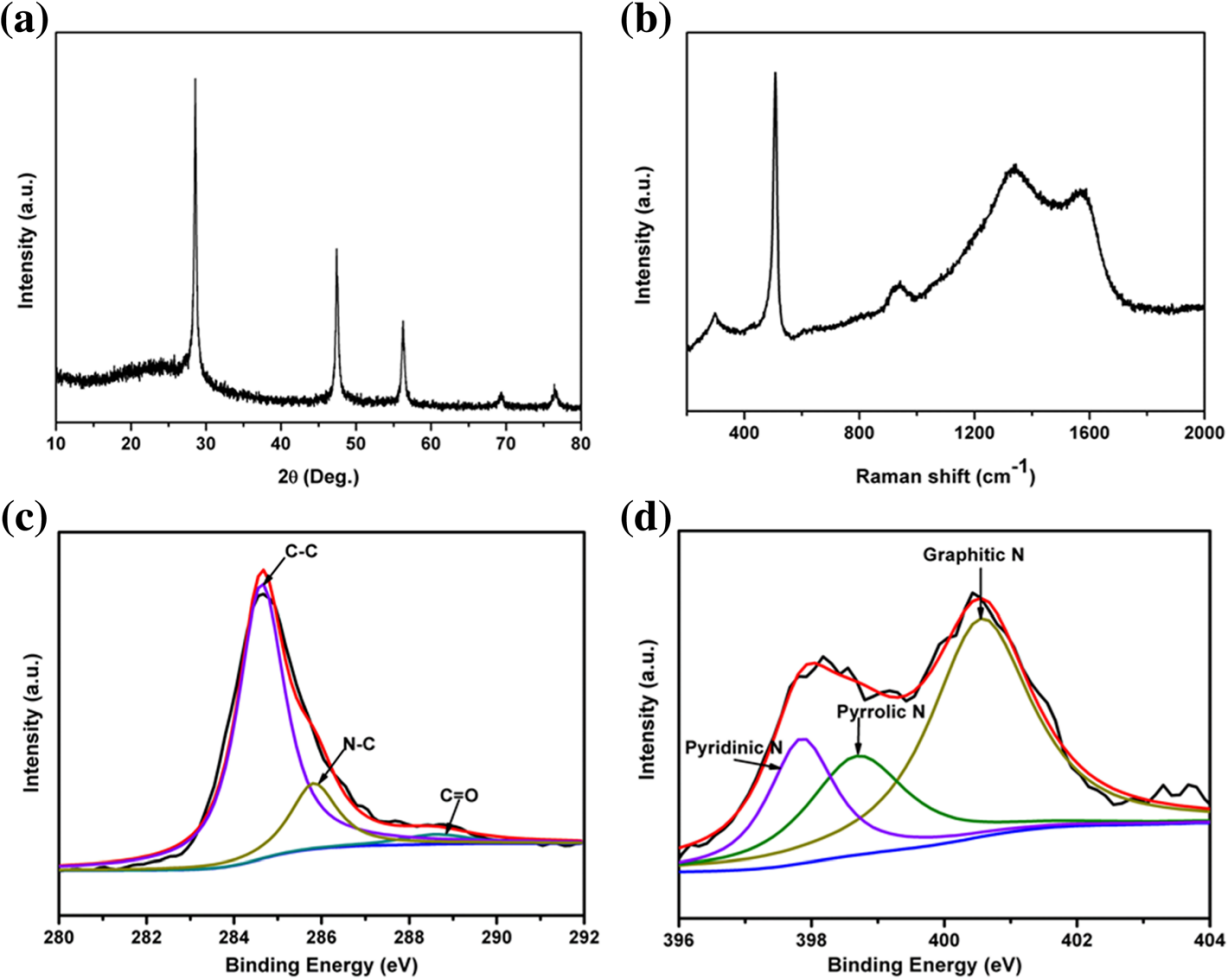
**a** PXRD patterns, **b** Raman spectra, **c** high-resolution C 1 s XPS spectra, and **d** high-resolution N 1 s XPS spectra of pSi@NC composite

To be used as LIB anode materials, the pSi were encapsulated with conductive polypyrrole to form pSi@NC composites. PXRD pattern of the pSi@NC composite showed an additional broad peak at around 23° (Fig. 3a), suggesting that the nitrogen-doped carbon layer is amorphous [39]. The Raman spectrum of the pSi@NC composite (Fig. 3b) showed two wide peaks at 1335 and 1585 cm^−1^ assigned to the D and G bands of graphitic carbon [47], respectively, which confirms the PXRD result. The intensity ratio of D band and G band (I_D_/I_G_) of the pSi@NC composite is about 1.07, implying a low degree of graphitization of the carbon layer. The C 1 s XPS spectra of pSi@NC showed the existence of N–C bond (285.85 eV in Fig. 3c), confirming that nitrogen was doped into the carbon framework [48]. The N 1 s XPS peak (Fig. 3d) can be divided into three peaks centralizing at 397.85, 398.72, and 400.57 eV, respectively, which belong to the pyridinic, pyrrolic, and graphitic types of nitrogen atoms doped in the carbon framework [39, 49]. The carbon content in pSi@NC composite was determined by TGA to be about 20 wt% (Additional file 1: Figure S3).

**Fig. 4:**
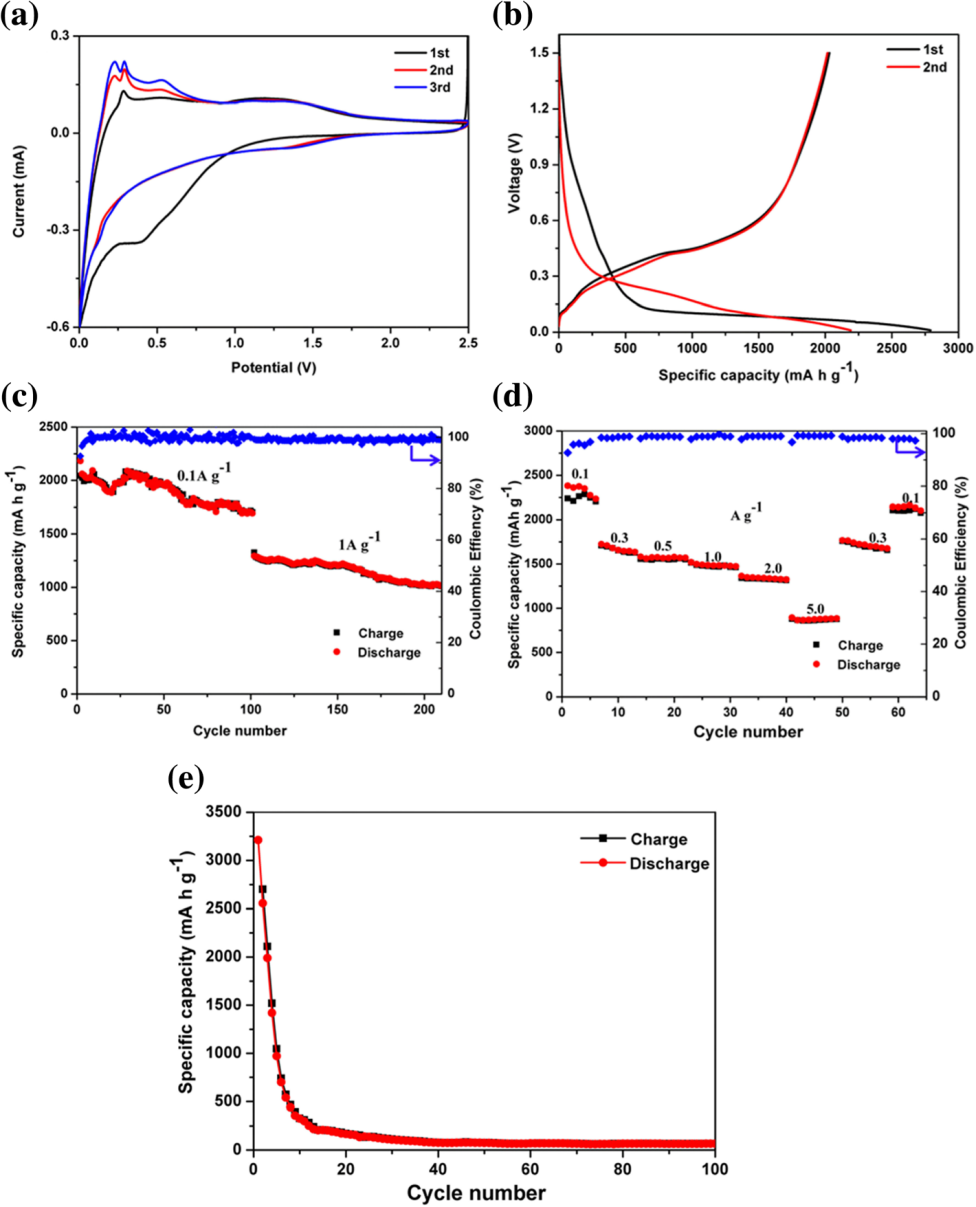
**a** CV curves, **b** charge-discharge curves, **c** long-term cycling performance at 0.1 A g^−1^ and 1 A g^−1^ for 100 cycles, respectively (current densities), and **d** rate performance cycled at various current densities of the pSi@NC composite electrode. **e** Cycling performance of commercial Si@NC composite at 0.1 A g^−1^ for 100 cycles

To characterize the electrochemical performances of the pSi@NC composite as the anode of LIBs, cyclic voltammetry (CV) measurements between and 2.5 V at a scanning rate of 0.2 mV s^−1^ were carried out. As shown in Fig. 4a, the first reduction peak around 1.5 V in CV curves was ascribed to the decomposition of the electrolyte additive (fluoroethylene carbonate FEC) [50]. The irreversible reduction peak was visible at the potential around 0.6 V during the first discharge and disappeared in the subsequent cycles, which was associated with the generation of the solid electrolyte interface (SEI) membrane [51]. The formation of SEI was due to the decomposition of organic solvents of electrolyte such as EC and DEC and led to the initial irreversible capacity loss [50, 52]. The peak near 0.1 V in the next CV curves represented the transition from crystalline silicon to amorphous Li_*x*_Si [53]. Meanwhile, during the charge process, two typical redox peaks at around 0.28 and 0.53 V were observed, which were related to the Li extraction process from Li_*x*_Si [54, 55]. Notably, the current intensities of both anodic and cathodic peaks gradually increased after the first cycles. This “activation” phenomenon should be mainly attributed to the gradual breakdown of the crystalline silicon structure [54, 56].

Figure 4b illustrates the first two discharge–charge curves of the pSi@NC composite anodes cycling at a current density of 0.1 A g^−1^. The pSi@NC composite had a long and flat discharge terrace around 0.1 V during the first discharge, which is in accordance with the characteristic terrace of the Li insertions of crystalline Si. The well-crystallized silicon turned amorphous and showed the representative charge/discharge profiles of amorphous silicon in subsequent cycles. The other potential plateaus which appeared around 0.6 V during the first lithiation process resulted from the SEI formation [57]. The results were in good agreement with the CV curves. The initial discharge and charge capacities were 2790 and 2036 mA h g^−1^, delivering a high initial Coulombic efficiency (CE) of 72.9%. The lower charge capacity could partly be due to the constraining effect of the oxide layer SiO_*x*_, which served as buffers to limit the volume expansion and extent of lithiation [58, 59]. Importantly, no obvious capacity decay was observed in the subsequent cycles, and the Coulombic efficiency was maintained nearly constant at around 100%.

Figure 4c shows the cycling performance of the pSi@NC composites anodes, which were conducted at a current density of 0.1 A g^−1^ for 100 cycles and at a current density of 1 A g^−1^ for subsequent 100 cycles. The pSi@NC nanocomposite anodes showed a capacity of 1720 mA h g^−1^ after 110 cycles at a current density of 0.1 A g^−1^, corresponding to a 79% capacity retention. Furthermore, the pSi@NC composite electrodes delivered a reversible capacity of 1010 mA h g^−1^ at 1 A g^−1^ after subsequent 110 cycles, with a capacity decay rate of 0.2% per cycle from 101 to 210th cycle. Figure 4d shows the rate performance of the pSi@NC electrode. The pSi@NC electrode achieved discharge capacities of 2360, 1690, 1570, 1470, 1320, and 850 mA h g^−1^ at the current density of 0.1, 0.3, 0.5, 1.0, 2.0, and 5.0 A g^−1^, respectively. The discharge capacity could be recovered to approximately 2160 mA h g^−1^ when the current density was returned back to 0.1 A g^−1^, proving that the pSi@NC composite anode had an outstanding electrochemical reversibility. In comparison, commercial silicon powder (Fig. 4e) coated with the conducting nitrogen-doped carbon as an anode reached a high initial discharge capacity of 3230 mA h g^−1^, but suffered severe capacity deterioration to 110 mA h g^− 1^ after 100 cycles at 0.1 A g^−1^. These result suggested that the conducting nitrogen-doped carbon layer and the porous structure in pSi@NC could provide the fast ion/electron transport pathways and maintained the structural stability, thus endowing the pSi@NC composite anode with good rate performance and excellent reversibility [21, 39, 60]. In addition, surface oxidation in pSi might also contribute to improve the cycling efficiency of lithium-ion batteries, which limited the volume expansion of the silicon particles and avoided some side reactions according to the previous studies [58].

## Conclusions

In summary, we developed a new method to prepare nanoporous silicon in high yields based on the reaction of magnesium silicide (Mg_2_Si) in acidic ionic liquid. When coated with the nitrogen-doped carbon layer and applied as an anode of lithium-ion battery, the obtained silicon-carbon composites exhibited high reversible capacity, long-term cycling stability, and high initial Columbic efficiency. The N-doped carbon-coating layer supplied the efficient conductive pathways for fast lithium-ion transportation and electron transfer, which is beneficial for enhancing the electrochemical properties of silicon particles. Since the reaction condition is relatively mild, and the yield of the products is over 82%, this preparation method could be extended to the mass production of silicon anode materials.

## Additional file

Additional file 1: **Figure S1.** PXRD patterns of the pristine products. **Figure S2** SEM images of pSi and commercial silicon. **Figure S3** TG curve of pSi@NC composite. (DOCX 730 kb)

## Abbreviations

[Bmim]Cl: 1-Butyl-3-methylimidazolium chloride
AlCl_3_: Aluminum chloride
CV: Cyclic voltammetry
EDS: Energy-dispersive spectroscopy
Mg_2_Si: Magnesium silicide
pSi: Porous silicon nanoparticles
pSi@NC: Nitrogen-doped carbon coated on porous silicon nanoparticles
PXRD: Powder X-ray diffraction
SEM: Scanning electron microscopy
TEM: Transmission electron microscopy
TGA: Thermogravimetric analysis
XPS: X-ray photoelectron spectroscopy
