# Supplementary material for: Ionothermal Synthesis of Crystalline Nanoporous Silicon and Its Use as Anode Materials in Lithium-Ion Batteries
Source: Nanoscale Res Lett. 2019 Jun 6;14:196. doi: 10.1186/s11671-019-3024-9 (PMC6554372; doi:10.1186/s11671-019-3024-9)
Supplement: Supplementary file 1 — Figure S1. PXRD patterns of the pristine products. Figure S2 SEM images of pSi and commercial silicon. Figure S3 TG curve of pSi@NC composite. (DOCX 730 kb) [file 11671_2019_3024_MOESM1_ESM.docx]

**Additional file 1**

Ionothermal Synthesis of Crystalline Nanoporous Silicon and Its Use as Anode Materials in Lithium Ion Batteries

Fei Wang, ^[a]^ BaoXun Zhao, ^[a]^ Wenwen Zi, ^[a]^ HongBin Du^[a*]^

***Electronic Supplementary Information contains Figure S1-S2***


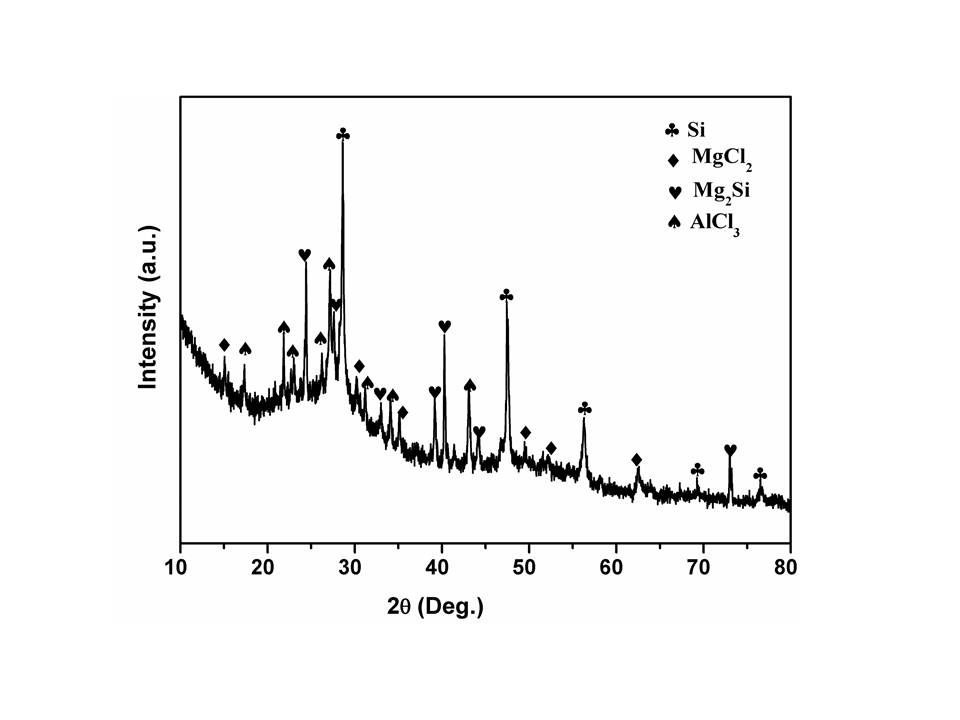


**Figure S1.** PXRD pattern of the pristine product.


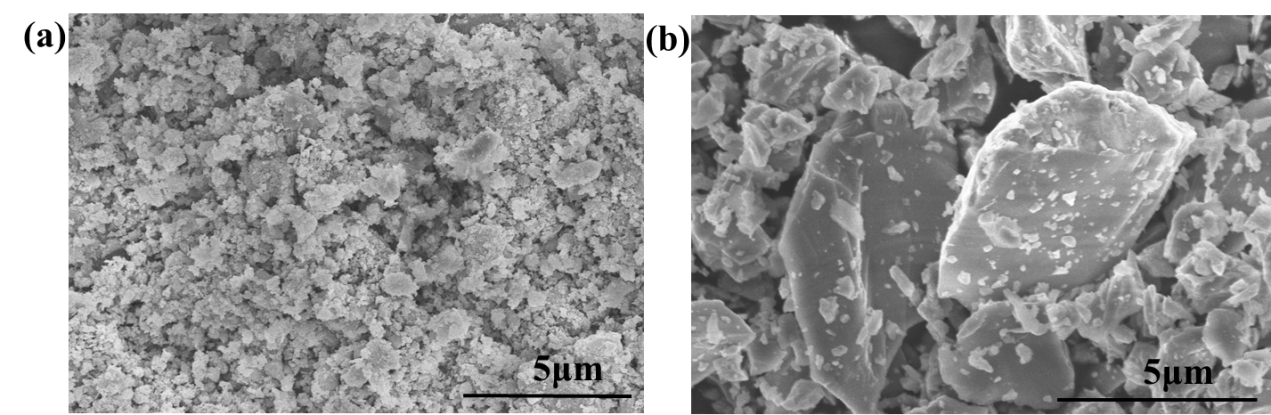


**Figure S2.** SEM images of (a) pSi and (b) commercial silicon.





**Figure S3.** TG curve of pSi@NC composite.
